# The Effect of Acupoint Application of Sinomenine for Rheumatoid Arthritis Measured by Microdialysis and UPLC-MS/MS

**DOI:** 10.1155/2019/5135692

**Published:** 2019-11-28

**Authors:** Rui Wang, Jing Yang, Yuxuan Liu, Bixi Guan, Qun Feng, Yanhong Wang, Shaowa Lv, Zhixin Yang, Yongji Li

**Affiliations:** ^1^College of Pharmacy, Heilongjiang University of Chinese Medicine, Harbin, Heilongjiang 150040, China; ^2^College of Basic Medical Science, Heilongjiang University of Chinese Medicine, Harbin, Heilongjiang 150040, China

## Abstract

**Objective:**

This study aimed to investigate the treatment effects of acupoint application of sinomenine in rheumatoid arthritis (RA). RA models were constructed using male New Zealand rabbits.

**Methods:**

The rabbits were randomly divided into a blank control group and four experimental groups as follows: ST 36 group (acupoint application of sinomenine at Zusanli); GB 34 group (acupoint application of sinomenine at Yanglingquan); knee-joint group (application directly at the site of the knee joint); and oral administration group (sinomenine administered by gavage). In all rabbits, microdialysis was applied at the knee joint to obtain samples. Pharmacokinetic (PK) and pharmacodynamic (PD) parameters were measured by ultraperformance liquid chromatography-tandem mass spectrometry (UPLC-MS/MS), and the PK/PD models were established according to the parameters derived.

**Results:**

Sinomenine concentration was in the range of 0.832–208 ng/mL, and the peak area showed a good linear relationship with the regression equation of *y* = 539.64*x* + 953.81; *r* = 0.9998. Moreover, good specificity and precision were obtained for the LC-MS/MS method of sinomenine evaluation in the microdialysate samples. The PK analysis showed that the sinomenine effect time was longer in the ST 36 group (area under the time-concentration curve (AUC): 12683.81 h·ng/ml and *T*_max_: 6.21 h) than in the other groups. Arginine and citrulline were selected as the indices for PD, and in the analysis of parameters for PK/PD, the highest value of *E*_max_ and the lowest value of *k*_e0_ were obtained in the ST 36 group.

**Conclusion:**

Acupoint application of sinomenine at ST 36 has potential for use in patients with RA by enabling enhanced and prolonged treatment effects.

## 1. Introduction

Rheumatoid arthritis (RA) is defined as chronic autoimmune inflammatory arthritis that causes erosion and destruction of the bone, degradation of the cartilage, swelling of the joint, and inflammation of the synovial membrane [1]. Reports have indicated that RA morbidity is approximately 1-2% worldwide, and that the disease is mostly prevalent in developed countries [2]. RA treatment is administered mainly to relieve symptoms, eliminate inflammation, and restore joint function. However, the precise etiology and pathophysiology of RA remain unclear, due to lack of knowledge of the disease origin and contraindications for long-term administration of several drugs, such as nonsteroidal anti-inflammatory drugs (NSAIDs), based on possible side effects [3]. Therefore, studies that help identify safe and effective therapeutic strategies for the treatment of RA are required.

*Sinomeniumacutum* is a traditional Chinese medicine that has been used in the acupoint treatment of RA for over hundreds of years in China, due to its beneficial effects with relatively few side effects; sinomenine is the main component of *Sinomeniumacutum* [4]. Sinomenine has been considered an ideal drug for the treatment of RA based on its immune-suppressive and anti-inflammatory activities [5]. However, high plasma concentrations of systemically-administered sinomenine hydrochloride may lead to histamine release, which can cause rashes and gastrointestinal side effects [6]. Topical delivery can increase the sinomenine hydrochloride concentration in the synovial fluid without concomitant high plasma concentrations, but the presence of the stratum corneum layer prevents efficient absorption [5], and hence administration routes with fewer side effects and high absorptivity are needed. Acupoint administration is a specialized therapy in Chinese medicine that is often used in traditional Chinese medicine to treat diseases such as frozen shoulder, allergic rhinitis, and lumbar disease [7–9]. Compared with other routes of administration, such as intravenous, muscular, and gastrointestinal, acupoint administration is safer because it can avoid some side effects. Among different acupoints, Zusanli (ST 36) and Yanglingquan (GB 34) are used commonly in modern treatment [10, 11]. Current studies have reported that acupuncture at ST 36 can promote anti-inflammation [12], inhibition of acute gastric mucosal injury [13], and suppression of chronic neuropathic pain [14]. Moreover, GB 34 has been shown to suppress chronic neuropathic pain [14] and to be a regulatory factor of motor-related network functions [15]. However, whether acupoint application of sinomenine at ST 36 and GB 34 can improve the therapeutic effect of sinomenine remains unclear.

Microdialysis is a new in vivo sampling technique that is applied to the study of pharmacokinetics and drug metabolism in the blood; it has the advantage of less tissue damage and provides more direct access to data [16]. In the present study, in order to investigate the effect of sinomenine on acupoints for treatment of RA, we constructed a rabbit model of RA, and collected samples from the knee using microdialysis; in addition, to establish the PK/PD models, we measured pharmacokinetic (PK) and pharmacodynamic (PD) parameters by ultraperformance liquid chromatography-tandem mass spectrometry (UPLC-MS/MS). The results obtained enable the development of a therapeutic strategy with sinomenine for the case management of RA.

## 2. Materials and Methods

### 2.1. Chemicals

Sinomenine was obtained from Pharmaceutical and Biological Products Inspection (batch number: 110774–200206, Shanghai, China). Ovalbumin was purchased from Boao Shanghai Biological Technology Co., Ltd. (batch number: 020902, Shanghai, China); Freund's complete adjuvant was supplied by SIGMA Co. (batch number: F5881, St. Louis, MO, USA); reference-substance solution was obtained from the China Offices of Pharmaceutical and Biological Products (batch number: 110774–200206, Shanghai, China); HPLC-grade acetonitrile was purchased from Symer Flight Technology Co., Ltd. (batch number: L650P75, China).

### 2.2. Animals

Six-month-old male New Zealand rabbits weighting 2.5–3 kg were obtained from the Heilongjiang University of Chinese Medicine Laboratory Animal Center (Certificate of Conformity SYXK (black) 2013-004). The rabbits were placed in separate cages and fed a standard diet for 1 week. The protocol was approved by the Ethical Committee for Biological and Medical Experimentation of Heilongjiang University of Chinese Medicine.

### 2.3. Preparation of Rheumatoid Arthritis Model Rabbits (RAMR) and Administration Route

To develop the experimental rabbit model of RA, the fur of the bilateral hind knees and the scapula region was removed with depilation agent, the areas were disinfected with alcohol, and procedures were performed as follows: on day 1, anesthesia was induced via 2% pentobarbital injections into the ear vein, and 1 mL of emulsion was injected at five points of the scapular area for sensitization. On day 29, 0.5 mL of ovalbumin solution (10 mg/mL) was injected at bilateral midpoints of the connecting lines between the highest point of the tibia and the lower margin of the iliac ligament. The success criteria of the RA model construction were based on the recommended human RA standards of the American College of Rheumatology and European League against Rheumatism and are summarized in Supplementary [Supplementary-material supplementary-material-1].

A total of 20 RAMR were randomly divided into a blank control (*N* = 4), an oral administration (*N* = 4), and a dermal medication (*N* = 12) group. The dermal medication group was then subdivided into a typical acupoint (ST 36) (*N* = 4), an atypical acupoint (GB 34) (*N* = 4), and a nonacupoint transcutaneous (knee joint) administration group (*N* = 4). Briefly, the oral administration group was administered sinomenine powder (400 mg/kg) by gavage, and the dermal medication groups were treated with sinomenine powder (400 mg/kg) by external application (as illustrated in Supplementary [Supplementary-material supplementary-material-1], the drug package was directly attached to the administration site).

Subsequently, microdialysate samples were collected at different time points, 30 minutes posttreatment and 1 to 14 hours posttreatment, at unit interval via microdialysis probes. All probe implantation methods are summarized in Supplementary [Supplementary-material supplementary-material-1]. After sample collection, 5 *μ*L of microdialysate was combined with 100 *μ*L chloroform solution in a 2 mL centrifuge, and vortexing was performed for 1 minute, followed by centrifugation at 4000 rpm for 10 minutes. Next, 120 *μ*L of the substratum solution was dried at 40°C. The residues were dissolved in 50 *μ*L methyl alcohol, and vortexing was performed for 3 minutes. Quantitative analysis was performed using a Waters UPLC-TQD triple-quadrupole mass spectrometer (Waters Technologies Limited, Shanghai, China). The reference-substance solution (RSS) was prepared with 2.08 mg sinomenine in 100 mL volumetric flasks.

### 2.4. Determination of Sinomenine in the Microdialysis Samples by UPLC-MS/MS

A chromatographic ACQUIT YUPLC® HSS T_3_ column (2.1 × 75 mm^2^; internal diameter, 1.8 *μ*m; Waters Technologies Limited, Shanghai, China) was used and maintained at 30°C. Isocratic chromatography was performed using a mobile phase of acetonitrile (10 mmol/L) and ammonium acetate (52 : 48, v/v) at a flow rate of 0.2 mL/min. During the quantitative analysis, electrospray ionization (ESI) parameters were set as follows: capillary voltage, 2.8 kV; cone voltage, 65 V; capillary temperature, 350°C; nitrogen flow velocity, 650 L/h and 50 L/h; and collision energy, 40 V. The ionization mode was positive with a parent ion of *m*/*z* 330 and a daughter ion of *m*/*z* 181.2.

### 2.5. Data Analysis of PK, PD, and PK/PD

PK parameters, such as the peak plasma drug concentration (*C*_max_) and the time to peak plasma drug concentration (*T*_max_), were read visually from the concentration-time profiles. Other PK parameters, such as the area under the time-concentration curve (AUC), absorption rate (*k*_01_), elimination rate (*k*_10_), half-life of absorption (*k*_01__HL), and half-life of elimination (*k*_10__HL), were obtained using the WinNonlin Examples Guide version 5.2.1 [17].

Arginine and citrulline were analyzed using an Ultimate® XB-C_18_chromatographic column (4.6 × 250 mm^2^; internal diameter, 5 *μ*m; Waters Technologies Limited, Shanghai, China). Mobile phase A was sodium acetate with acetonitrile (pH 6.5; 93 : 7, v/v), and mobile phase B was water with acetonitrile (94 : 6, v/v). Moreover, the flow rate was 1 mL/min at a wavelength of 254 nm. For the PD analysis of microdialysis samples, 50 *μ*L of the samples was added to a 2 mL centrifuge tube, with 25 *μ*l of derivative reagents I and II. Vortexing was performed for 1 minute, and 200 *μ*L n-hexane was added to each of the samples. The effect value (*E*) was calculated according to the following formula

(1) $$\begin{array}{c} E=\text{IR}\left(\%\right)=\frac{C_{o}-C_{t}}{C_{o}}\times 100\%, \end{array}$$

where IR (%) is the inhibition rate and *C*_*o*_ and *C*_*t*_ are the initial concentration and the measured value, respectively.

The PK/PD model was constructed according to the WinNonlin Examples Guide software version 5.2.1 [18]. The effect compartment drug concentration (*C*_e_) was calculated using the following formula:

(2) $$\begin{array}{c} C_{\text{e}}=\frac{Dk_{\text{e0}}k_{01}}{V_{\text{c}}}\times \left[\frac{e^{-k_{10}t}}{\left(k_{\text{e0}}-k_{10}\right)\left(k_{01}-k_{10}\right)}+\frac{e^{-k_{01}t}}{\left(k_{\text{e0}}-k_{01}\right)\left(k_{10}-k_{01}\right)}+\frac{e^{-k_{\text{e0}}t}}{\left(k_{01}-k_{\text{e0}}\right)\left(k_{10}-k_{\text{e0}}\right)}\right], \end{array}$$

where *D* is the dose; *V*_c_ is the volume of the effect compartment; and *k*_01_ and *k*_10_ are the absorption rate and elimination rate, respectively.

S-*E*_max_ was calculated according to the following formula:

(3) $$\begin{array}{c} E=\frac{E_{\max}\times C_{\text{e}}}{\left(C_{\text{e}}+{\text{EC}}_{50}\right)}, \end{array}$$

where *E* is the pharmacological effect; *E*_max_ is the calculated maximum effect; and EC_50_ is the drug concentration in the effect compartment yielding half of the maximal effect.

## 3. Results

### 3.1. Quantitative Analysis of Sinomenine by UPLC-MS/MS

To prepare the standard curve for sinomenine, appropriate amounts of RSS were added to physiological saline to achieve concentrations of 0.832 ng/mL, 2.08 ng/mL, 4.16 ng/mL, 12.48 ng/mL, 24.96 ng/mL, 41.6 ng/mL, 124.8 ng/mL, and 208 ng/mL. Standard solutions were filtered with a 0.22 *μ*m Millipore filter, and the standard curve of the samples was obtained by UPLC-MS/MS.

**Figure 1:**
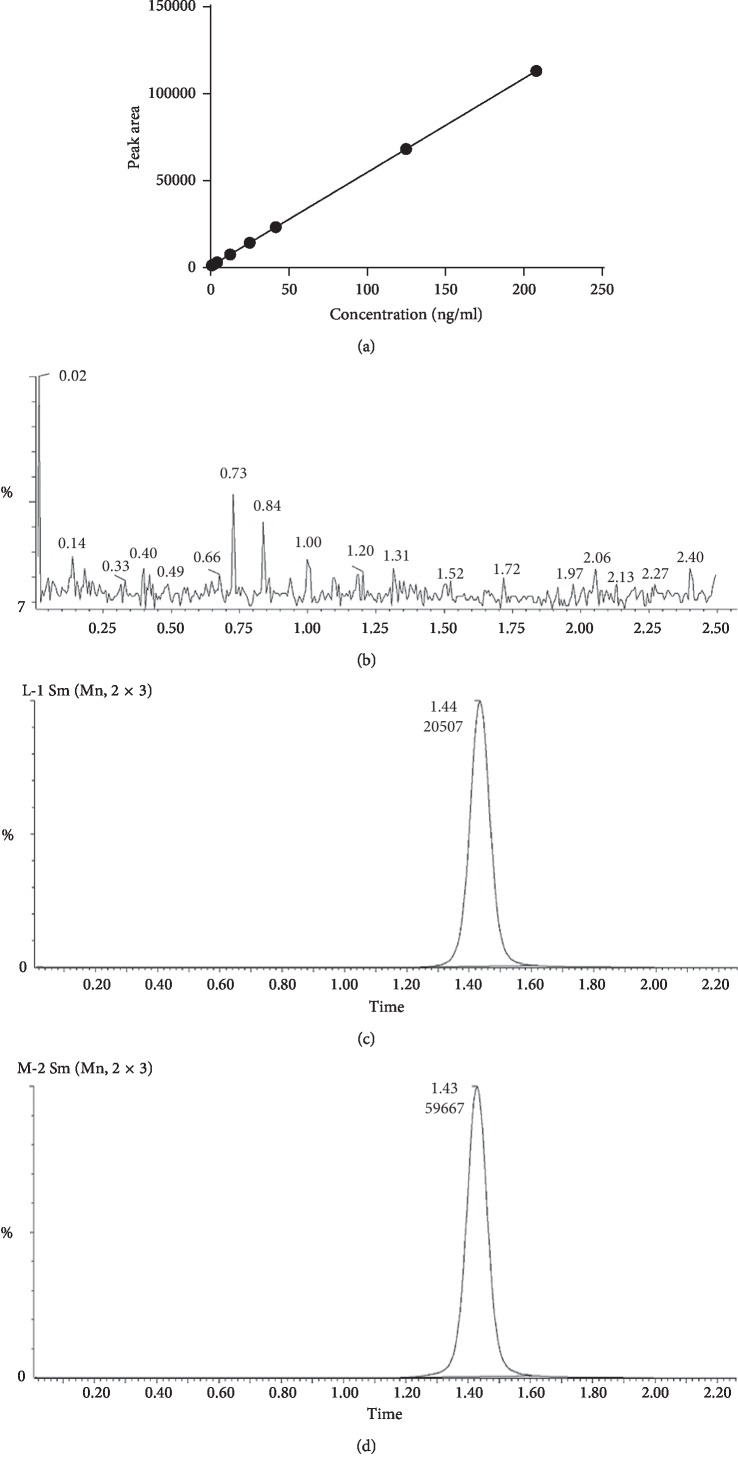
Determination of the specificity, precision, and accuracy of the measurement values of HPLC-MS/MS of sinomenine in microdialysate samples. (a) Standard curve of sinomenine. (b) Chromatogram of the blank solution. (c) Chromatogram of sinomenine RSSs. (d) Chromatogram of the MD sample with sinomenine.

The sinomenine concentration was plotted along the *x*-axis, against the peak area at each concentration on the *y*-axis, yielding the linear regression equation *y* = 539.64*x* + 953.81 (*r* = 0.9998) (Figure 1(a)). The regression analysis showed a good linear relationship between the peak area and the sinomenine concentration in the range of 0.832–208 ng/mL.

The chromatograms of the blank saline solution, control solution, and microdialysis solution samples are shown in Figures 1(b)–1(d). The MS analysis showed no impurity peak at the peak position of sinomenine, and the peak shape of sinomenine was good. For the precision and accuracy evaluation, three different sinomenine concentrations of RSS including 0.832 (low), 24.96 (middle), and 124.8 (high) ng/mL were selected as quality control samples for further analysis. The intraday CVs of the low, middle, and high concentration RSSs were 3.14%, 2.22%, and 1.36%, respectively, and the interday CVs were 4.79%, 2.55%, and 1.51%, which indicates that the precision of the instrument was up to standard levels.

### 3.2. Detection of Local PK Indices of Sinomenine

**Figure 2:**
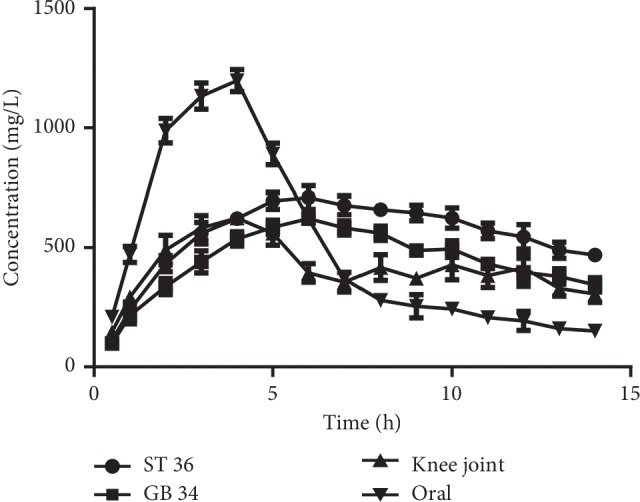
Mean concentration-time curves of sinomenine. The rabbits were randomly divided into the blank control group, the ST 36 group, the GB 34 group, the knee-joint group, and the oral group.

The analysis of the concentration of sinomenine in the articular cavity of the five groups was based on the microdialysis samples (Supplementary [Supplementary-material supplementary-material-1]). The values of the PK parameters for the different administration groups are shown in Table 1. In the oral administration group, absorption of sinomenine was observed at a *C*_max_ of 1106.87 ng/mL at 2.78 h; in the nonacupoint group, absorption of sinomenine was observed at a *C*_max_ of 534.59 ng/mL at 3.23 h; in the ST 36 and GB 34 groups, similar PK trends were observed, and values of *C*_max_ were 694.76 ng/mL at 6.21 h and 578.61 ng/mL at 5.89 h, respectively. Specially, the drug-time curve of the ST 36 group was gentler compared with the curves of the other groups (Figure 2). Moreover, although the absorption of sinomenine in the body after percutaneous administration happened at a slow rate, the absorption duration was higher than after oral administration. Moreover, among the percutaneous administration groups, the ST 36 group was superior to the GB 34 group and the knee-joint group, showing faster absorption rates and longer effect times of sinomenine.

### 3.3. PD Analysis

**Figure 3:**
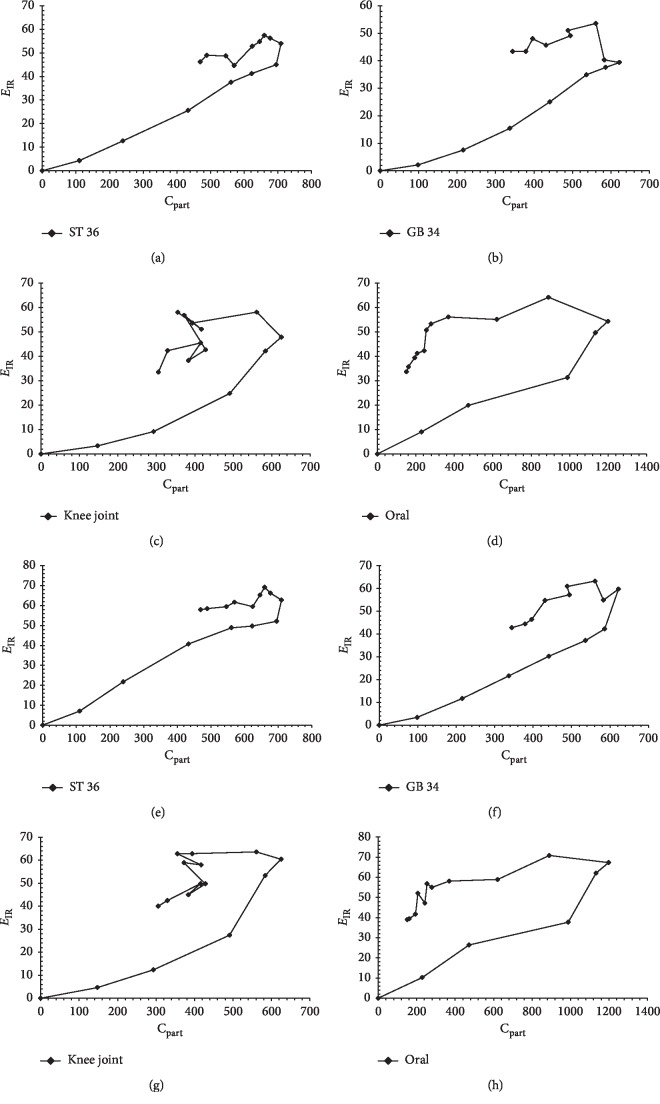
Drug concentration curves and pharmacological effects for the four administration routes (ST 36, GB 34, knee joint, and oral). (a)–(d): inhibition rate of arginine; (e)–(h): inhibition rate of citrulline.

The effect curves of drug concentration (*C*_p_) and inhibiting percentage (*E*_IR_) of arginine and citrulline are shown in Figures 3(a) and 3(b), respectively. The results of the PD analysis of the four different administration routes show that the relationship between the pharmacological effect (inhibition rate of citrulline or arginine) and the sinomenine dialysis concentration was best fitted by a counterclockwise curve. Briefly, the pharmacological effect of sinomenine was maintained at a high level, and a peak effect was observed even during the period of microdialysis concentration decrease, which indicates the presence of an effect compartment.

### 3.4. PK/PD Analysis

The PK and PD parameters of the four administration routes were entered into a fitting process with WinNonlin. As shown in Table 2, for PK/PD parameters of arginine, the *E*_max_ of the ST 36 group, at 84.7% was higher than that of the oral administration group, at 83.42%, the GB 34 group, at 79.91%, and the knee-joint group, at 65.9%. This indicates that ST 36 administration led to higher efficacy than oral administration, and that, with regard to the percutaneous administration routes, ST 36 and GB 34 acupoint administration leads to greater inhibition of arginine than nonacupoint (knee-joint group) administration. Moreover, a lower value of EC_e50_ was obtained in the knee-joint group than in the other groups, suggesting a stronger binding force with the receptor in the knee-joint group, which may be related to the direct application of the treatment above the target-knee cavity. Regarding *k*_e0_ via other administration routes, *k*_e0_ was highest in the oral group and lowest in the ST 36 group, indicating that sinomenine in the ST 36 group had a low equilibrium rate between the virtual effector and target organ, and that the elimination rate of the effector organ was slower in this group than in the oral administration group. The PK/PD analysis of citrulline showed results similar to those obtained for arginine.

**Figure 4:**
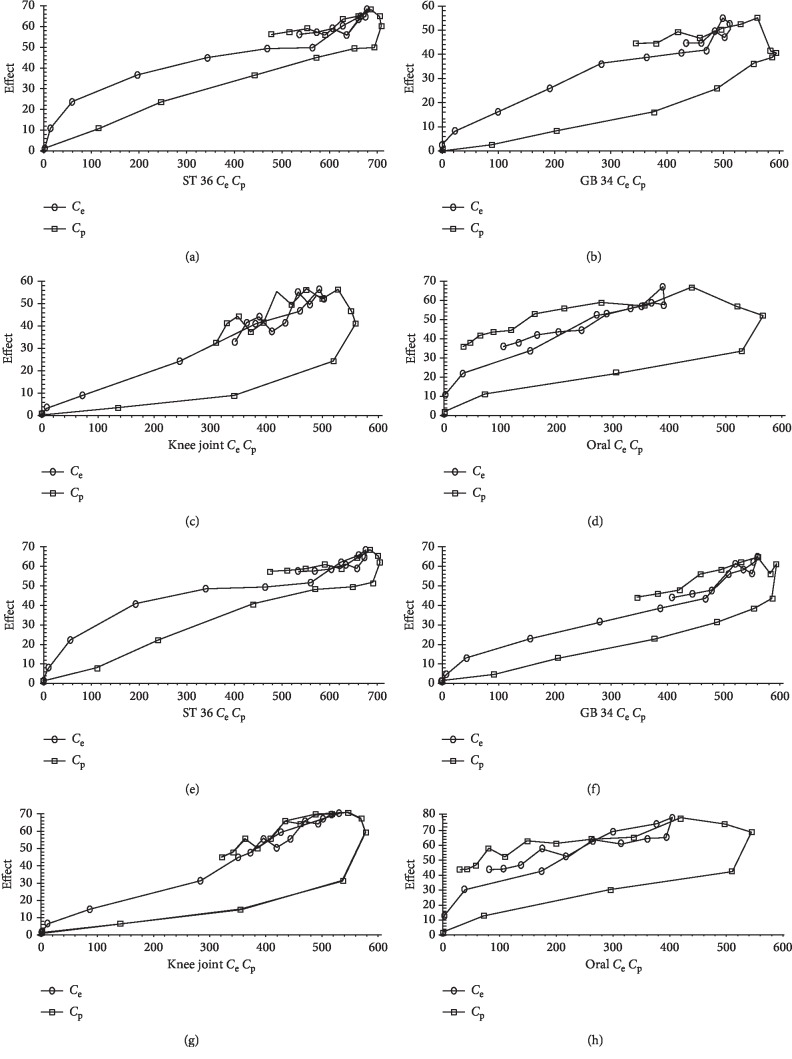
The *C*_e_-effect curves and *C*_p_-effect curves of arginine and citrulline in the different groups. (a)–(d): The curves of arginine for the ST 36, GB 34, knee-joint, and oral administration groups. (e)–(h) The curves of citrulline for the ST 36, GB 34, knee-joint, and oral administration groups.

Next, the fitting curves of the sinomenine concentration-effect were plotted for the four administration routes. There were no overlaps between the *C*_e_-effect curves and the *C*_p_-effect curves (Figure 4), and the concentration-effect curve showed a good correlation, suggesting that the fitting effect was optimal.

## 4. Discussion

To the best of our knowledge, the current study is the first to investigate the effect of sinomenine applied at acupoints for the treatment of RA by microdialysis and UPLC-MS/MS. Our analysis of the PK and PD of sinomenine applied at the acupoints ST 36 and GB 34, at the knee joint, and via oral administration for RA treatment, as well as the proposed PK/PD model reveals a high absorption degree of sinomenine and a low elimination rate of sinomenine in the ST 36 group and thus suggests that the application of sinomenine at ST 36 has potential benefits in the treatment of patients with RA. Moreover, this study analyzed the changes in drug concentrations and effects in the articular cavity, in order to elucidate the mechanism of action of sinomenine against RA.

Due to the small volume of microdialysis samples and the unavoidable dilution of the samples during collection, HPLC-MS has become the preferred method, combined with microdialysis technology, due to its high sensitivity and low requirements for sample size [19, 20]. In the current study, we determined the specificity, precision, and accuracy of sinomenine in microdialysis samples by UPLC-MS/MS. The standard curve of sinomenine showed a good linear relationship, and similar peaks without impurities were found in the MS of the sinomenine RSSs and microdialysis samples. Collectively, our results indicate good sensitivity, reliability, and robustness of the method for the determination of sinomenine in microdialysis samples, as demonstrated in our rabbit model.

Traditional administration routes of sinomenine have several limitations, such as side effects and low absorption rates. Previous studies have proposed several methods to resolve the problem, such as optimizing control release with electroporation, adding bacterial cellulose to the formulation, and drug delivery using elastic liposomes [10, 21, 22]. However, such methods have limitations regarding high costs and complex operations. In the present study, we investigated the effects of sinomenine after percutaneous acupoint administration (ST 36 and GB 34) using the PK/PD model and found that ST 36 administration was the best route of percutaneous administration, showing the longest duration of drug effects. An increasing number of studies have revealed the role of acupoint administration for the treatment of different diseases, such as chronic gastritis of gastric blood stasis type [23], acute lumbar sprain [24], and functional dyspepsia [25]. Among a variety of different acupoints, ST 36 and GB 34 are used commonly in modern treatment [10, 11]. Specially, acupuncture at ST 36 has been reported to promote anti-inflammation effects [12], inhibit acute gastric mucosal injury [13], and suppress chronic neuropathic pain [14]. Moreover, ST 36 plays a role in the treatment of diseases, in accordance with our results. Overall, our results demonstrate that the application of sinomenine at ST 36 is more beneficial for the treatment of RA than other application sites or administration routes, which may be due to the fact that RA is an inflammatory disease.

PK enables an investigator to acquire a clear understanding of the characteristics of drug action, to design new drugs, and to ensure rational drug use based on studies focused on dynamic regulation of drugs in vivo; therefore, a PK analysis of drugs should be conducted in animal models for the development of therapy in humans [26, 27]. Some studies have investigated the PK profile of sinomenine in rats after oral administration [28, 29] or intramuscular injection [30]. However, to the best of our knowledge, our study is the first to report the findings of a PK analysis of sinomenine after acupoint administration. Our results indicate that, although the oral group showed rapid absorption of sinomenine, the effect lasted for the shortest time in this group; the sinomenine effect time of the ST 36 group, in contrast, was longer than that in the oral, GB 34, and knee-joint group. According to the obtained *k*_01_ values, the knee-joint group showed the most rapid absorption of sinomenine, may be due to the fact that the treatment site was closest to the target site in the knee-joint group. Based on the results of our PK analysis, we suggest that sinomenine application at ST 36 may be effective in the treatment of RA.

NO is an important inflammatory index, due to its high levels in the serum and synovial fluid of patients with RA [31]. However, the detection of NO is difficult due to its gaseous state, and we hence focused on PD parameters of arginine and citrulline, which are important amino acids in the NO metabolic cycle. The PK/PD model parameter evaluation results showed the highest value of *E*_max_ in the ST 36 group, indicating that acupoint administration at ST 36 may amplify the inhibition of arginine. With regard to acupoint applications at either ST 36 or GB 34, we observed differences in *E*_max_ values, which indicate that clinicians should consider the administration of the disease treatment at the correct acupoint. *k*_e0_ is an important parameter in the PK/PD model, reflecting the speed of the drug elimination from the effector chamber as well as the rate of correspondence between the drug blood concentration and the effect [32]. In the present study, the ST 36 group showed a lower *k*_e0_ than the other groups, indicating that acupoint application at ST 36 leads to the longest effect and is thus the most efficient among all administration routes.

## 5. Conclusion

We find that acupoint application of sinomenine at ST 36 shows better effects in the treatment of RA, by enhancing and prolonging the drug effect of sinomenine, than application at GB 34, direct application at the site of the knee joint, or oral administration through gavage in the RAMR. Studies using PK/PD models that include larger samples are needed to verify the current results.

## Figures and Tables

**Table 1:** Pharmacokinetic parameters of sinomenine for the four administration routes.

Pharmacokinetic parameters	Values			
	ST 36	GB 34	Knee joint	Oral
*k*_01_ (1/h)	0.25	0.17	1.04	0.41
*k*_10_ (1/h)	0.1	0.18	0.06	0.42
AUC (h·ng/mL)	12683.81	9046	11165.71	7229.01
*k*_01__HL (h)	2.78	4.1	0.66	1.69
*k*_10__HL (h)	6.82	3.87	12.01	1.64
Tag (h)	0.13	0.15	0.28	0.38
*D*_0_/V (ng/mL)	257.7	323.9	128.9	274.9
*T*_max_ (h)	6.21	5.89	3.23	2.78
*C*_max_ (ng/mL)	694.76	578.61	534.59	1106.87

*C*_max_: peak plasma drug concentration; *T*_max_: time to peak plasma drug concentration; AUC: area under the time-concentration curve; *k*_01_: absorption rate; *k*_10_: elimination rate; *k*_01__HL: half-life of absorbed fraction; *k*_10__HL: half-life of eliminated fraction.

**Table 2:** Pharmacokinetic/pharmacodynamic parameters of arginine and citrulline in four administration routes.

PK/PD parameters	Arginine				Citrulline			
	ST 36	GB 34	Knee joint	Oral	ST 36	GB 34	Knee joint	Oral
*E*_max_ (%)	84.7	79.91	65.9	83.42	80.29	57.22	78.69	78.36
EC_e50_	246.77	181.56	169.89	357.43	195.89	307.4	113.5	207.53
*k*_e0_	0.6	0.69	0.67	0.74	0.6	0.66	0.7	0.72

## Data Availability

The datasets used and/or analyzed during the current study are available from the corresponding author on reasonable request.

## Abbreviations

RA: Rheumatoid arthritis
RAMR: Rheumatoid arthritis model rabbits
ESI: Electrospray ionization
RSS: Reference-substance solution
PK: Pharmacokinetics
PD: Pharmacodynamics
UPLC-MS/MS: Ultraperformance liquid chromatography-tandem mass spectrometry.

## Additional Points

(1) Sinomenine showed longest effect after application at acupoint ST 36; (2) LC-MS for sinomenine detection showed good specificity and precision; and (3) ST 36 acupoint application of sinomenine has potential benefits for the treatment of RA.

## Supplementary Materials

Supplementary Materials Supplementary table 1: criteria for the RA model construction; supplementary table 2: methods of probe implantation; supplementary table 3: the concentration of sinomenine in different groups at 14 h; supplementary figure 1: the drug package of sinomenine. The drug package was directly attached to the administration site.
