# Supplementary material for: The Effect of Acupoint Application of Sinomenine for Rheumatoid Arthritis Measured by Microdialysis and UPLC-MS/MS
Source: Evid Based Complement Alternat Med. 2019 Nov 28;2019:5135692. doi: 10.1155/2019/5135692 (PMC6907056; doi:10.1155/2019/5135692)

Supplementary Table 1. Criteria for the RA model construction

Index	Changes
Posture	Apathetic, activities decrease and slowly, joint deformation and cannot be loaded
Body temperature	Body temperature is gradually increased
Weight	Body weight is gradually increased after sensitization, but the rate of increase is gradually slow down
Diet	Diet reduced after each sensitization and then gradually restored
Joint diameter	Joint diameter is swollen obviously at the day of sensitization, and then restores to about twice the original

Supplementary Table 2. Methods of probe implantation

**Steps**	**Processes**
1 step	Rotatingly insert No. 5 injection needle to the place of drug delivery
2 step	Send a tear tube from the needle into the place of drug delivery
3 step	Pull out the needle and make the tear tube stay at the articular cavity
4 step	Microdialysis probe was inserted into the tear tube along the center of the tear tube
5 step	Tear the tear tube to both sides and pull it out simultaneously
6 step	Make sure that the probe was stayed in the articular cavity

Supplementary Table 3. The concentration of sinomenine in different groups at 14 h

Time	**Sinomenine concentration （ng/mL; $\bar{x} \pm s$, n=4）**			
	**ST 36 group**	**GB 34 group**	**Knee-joint group**	**Oral group**
0.5 h	110.07±22.68	98.5±24.42	147.32±10.12	208.85±25.12
1 h	239.26±32.72	215.85±34.79	292.84±20.30	472.10±33.74
2 h	433.55±34.09	336.77±33.99	490.89±62.10	988.20±51.39
3 h	560.93±45.01	440.84±46.53	583.52±51.32	1132.75±55.67
4 h	622.37±29.33	536.32±30.96	624.94±27.46	1198.26±46.32
5 h	695.40±35.32	586.12±29.99	560.79±50.71	890.02±45.66
6 h	709.73±49.70	622.07±28.87	394.04±38.57	621.20±21.77
7 h	677.09±38.83	582.81±30.71	355.57±41.20	369.08±24.11
8 h	659.99±18.88	561.02±29.90	417.10±54.10	279.05±24.67
9 h	645.95±33.36	488.70±28.39	372.40±27.23	253.81±49.07
10 h	624.08±42.21	494.60±35.70	428.10±61.98	242.55±29.90
11 h	570.22±33.10	431.27±28.86	383.57±49.88	206.32±23.49
12 h	545.99±51.76	396.25±59.52	415.53±60.20	193.73±40.01
13 h	488.94±34.43	378.99±32.65	329.09±32.21	160.81±25.65
14 h	469.23±24.45	344.32±29.87	305.43±35.65	150.99±25.89

Supplementary Figure 1. The drug package of sinomenine. The drug package was directly attached to the administration site

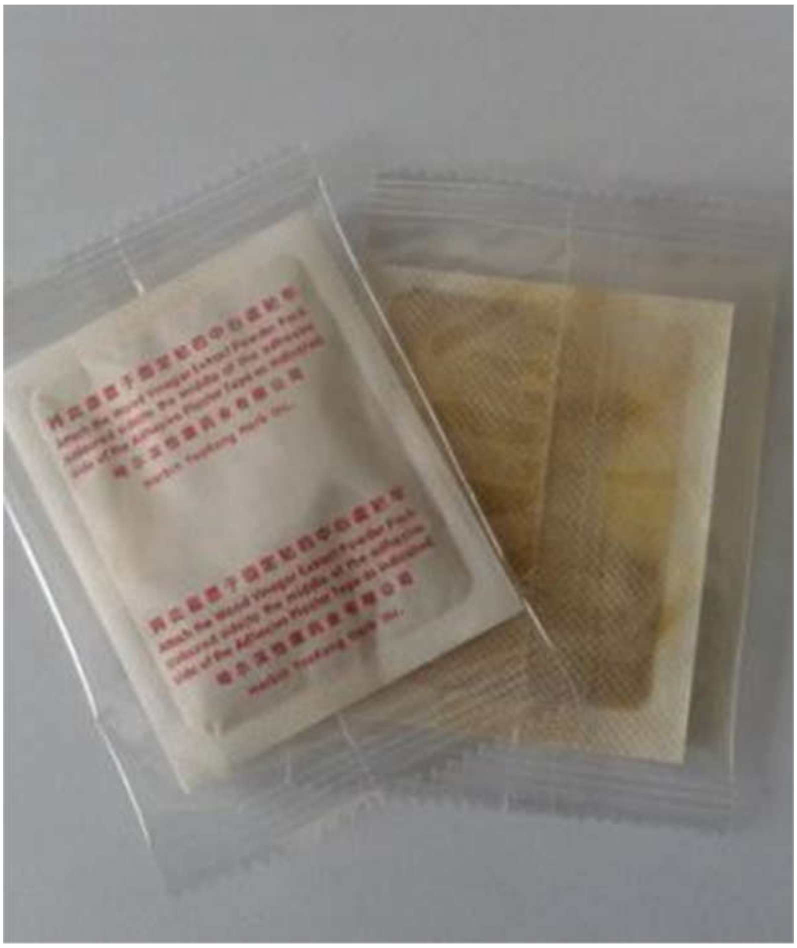

Supplement: Supplementary Materials — Supplementary table 1: criteria for the RA model construction; supplementary table 2: methods of probe implantation; supplementary table 3: the concentration of sinomenine in different groups at 14 h; supplementary figure 1: the drug package of sinomenine. The drug package was directly attached to the administration site. [file 5135692.f1.pdf]
